# A Localized Transient-Based Fault Location Scheme for Distribution Systems

**DOI:** 10.3390/s22072723

**Published:** 2022-04-01

**Authors:** Navid Bayati, Lasse Kappel Mortensen, Mehdi Savaghebi, Hamid Reza Shaker

**Affiliations:** 1 Centre for Industrial Electronics, Department of Mechanical and Electrical Engineering, University of Southern Denmark, 6400 Sønderborg, Denmark; mesa@sdu.dk; 2 Center for Energy Informatics, Maersk Mc-Kinney Møller Institute, University of Southern Denmark, 5230 Odense, Denmark; lkmo@mmmi.sdu.dk (L.K.M.); hrsh@mmmi.sdu.dk (H.R.S.)

**Keywords:** distribution system, fault location, overcurrent relay

## Abstract

Many distribution systems have several branches with only one protection system at the upstream system. This characteristic degrades the performance of traditional fault location schemes. In this paper, a localized fault location method based on the transient behavior of fault currents by using local data is proposed. The proposed scheme uses only local current and the voltage of the upstream overcurrent relay as input data of the fault location scheme. The formulation considers fault resistance, loads, and different fault locations. Furthermore, due to the usage of transient fault current data, the proposed method locates the fault within several milliseconds with a suitable range of error. To validate the effectiveness of this method, field measurement data, obtained from a real distribution system in East Jutland, Denmark operated by Dinel A/S, are used, and extensive real-time simulations are performed. The results prove that the proposed method locates different types of faults within an appropriate time and error, which can improve the maintenance and reliability of distribution systems.

## 1. Introduction

Faults in distribution systems cause undesirable disturbances in the standard operating condition. In distribution systems equipped with underground cables, faults can be caused due to different reasons, such as physical damage, and decay in the insulation of cables [1]. These unexpected faults in distribution systems deteriorate the power quality and system reliability and damage the power supply [2]. Therefore, developing fault location schemes is one of the great interests of electric utilities, as they help to estimate the required place for maintenance and reinforcement and support power restoration [3].

Typically, the fault location methods in distribution systems are divided into impedance-based and transient methods [4]. Like distance relay in high-voltage transmission lines, these methods use the estimation of fault resistance seen from the sensor unit to determine the faulty point [5]. In [6], the direct three-phase circuit analysis is used to locate the faults; however, the accuracy of this method is not evaluated for different fault conditions, such as different fault resistances. An impedance-based method based on the data from one end of the distribution system is presented in [7] to locate the fault in overhead lines. However, due to using the model of overhead lines, this method cannot be implemented in distribution systems with underground cables.

Transient-based fault location methods use the transient characteristics of voltage and current to estimate the fault distance [8]. In [9], the fault location and resistance are estimated by using features such as peak time and the magnitude of the fault current at the local point. The results show higher accuracy and speed compared with other impedance-based methods. The application of wavelet for locating faults in distribution systems is presented in [10], and it is shown that faults can be located within several milliseconds. However, this method is not able to locate faults in a system with multiple branches. In [11], the application of a support vector machine (SVM) for locating the fault is suggested. In this method, the slope of the fault current and magnitude are utilized to train the model by using simulated data. However, in practical systems, it would be impossible to access a high number of data to train the model of SVM and using the simulated data will cause some differences in the results of fault location scheme implementation.

Currently, the most common method for estimating the fault location is based on manual outage mapping using information from consumers [12]. However, due to the recent developments in monitoring systems, novel fault location methods in distribution systems have been suggested. In [13], the fault location is determined by using deep graph convolutional networks. In this method, the data from multiple sensors at different buses are used in the graph network model to locate the fault. The installation of phasor measurement units (PMU) in the distribution system for developing fault location identification is suggested in [14]. This method uses real-time data from PMUs and locates the fault based on state estimation. However, it requires a high-bandwidth communication channel and extra costs for the installation of additional equipment. The necessity of monitoring systems for fault location methods in distribution systems is highlighted in [15], and a modern monitoring infrastructure with a limited number of monitoring devices in the distribution system is developed to accurately locate the faults.

In contrast, in practical systems, only the current and voltage at the upstream power substation are monitored, and by calculating the impedance between the faulty point and substation, the fault location can be estimated [16]. Generally, in these cases, in which only the power substation is monitored, the existing methods estimate the fault location by neglecting the fault resistance and load impact during the fault. In [17] and [18], the fault location problem is formulated by the pattern recognition method; however, this method requires large databases including fault cases for the understudy distribution system. Therefore, in addition to requiring accurate modeling of the system, there is always the possibility of having different results during the test in real distribution systems. A traveling wave-based method is presented in [19] for fault location in a radial distribution system. However, this method requires the implementation of additional equipment and suffers from low accuracy in practical conditions due to the high number of laterals in distribution systems.

In this context, this paper proposes a scheme based on the current and voltage sensors at the main substation 10 kV feeder to locate the faults in the underground cable of a medium voltage (MV) distribution system. To avoid the installation of any additional sensor devices, the current and voltage waveforms measured by an overcurrent relay at the head end of the MV distribution system are applied. The peak time and value of the current are used as the main transient features for estimating the fault. Moreover, the impact of fault resistance and loads are considered in this method to provide a more accurate scheme. The proposed scheme is tested on a distribution system through comparison with real field data and performing real-time simulation using an OPAL-RT platform. The main innovation aspects of the proposed scheme are as follows:

(i). It provides a local fault location method, and no communication link or central monitoring system is required.
(ii). It locates the fault within several milliseconds thanks to using the transient features of the current and voltage.
(iii). It allows finding the fault location on branches and not only at buses thanks to the proposed scheme.

The rest of this paper is organized as follows: The characteristics of faults in distribution systems are investigated in Section 2. In Section 3, the fault location scheme based on the fault transient features and detailed framework of the proposed scheme is explained. The real-time simulation results of the proposed scheme are described in Section 4. Finally, the conclusions of the paper are presented in Section 5.

## 2. Fault Characteristics in Distribution Systems

**Figure 1:**
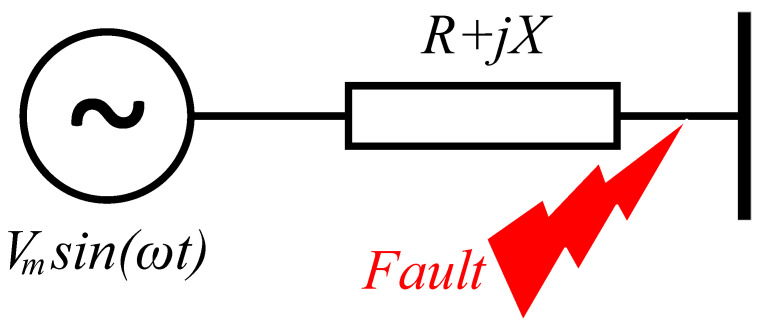
Fault current of a distribution system.

This section presents the calculation of fault current and its features in an AC distribution system when the decaying components of the fault current are also involved. Let us consider a sinusoidal 50 Hz power source, *V_m_sin*(*ωt*), connected to a distribution line with line impedance of *Z = R + jX,* where *V_m_* is the peak of source voltage, *ω* is the angular frequency, and *X* and *R* are the reactance (*Lω*) and resistance of the line, as shown in Figure 1. Therefore, the equation of the circuit during a fault can be written as:

(1) $$L\frac{di}{dt}+Ri=V_{m}\sin(\omega t)$$

where *L* is the inductance of the line. Therefore, by solving (1), the fault current time-domain equation can be determined as follows:

(2) $$\left\{ \begin{array}{l} i=\frac{V_{m}}{\sqrt{\left(R^{2}+L^{2}\omega^{2}\right)}}(\sin(\omega t-\theta )+\sin(\theta )e^{-\alpha t})\\ \theta =\tan^{-1}(\frac{L\omega }{R})\\ \alpha =\frac{R}{L}\\ \omega =2\pi f \end{array}\right.$$

In distribution systems, the value of *X/R* is low compared with that of higher voltage power systems, and it is in the range of 2–8 [20]. As shown in (2), the fault current is made by the summation of a sinusoidal and an exponential time-varying part, where the exponential part is defined as the DC decaying transient of the fault current. A physical explanation of the DC decaying transient during a fault in distribution systems is due to the high value of the inductance component of the impedance.

**Figure 2:**
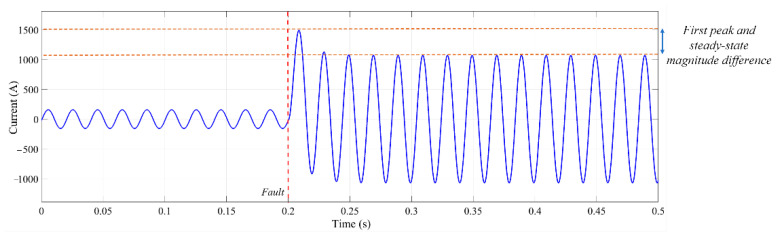
Fault current of a distribution system.

A fault current waveform of an AC distribution system, extracted by simulation, can be observed in Figure 2. As can be seen from Figure 2, the fault current magnitude is several times higher than the rated current. Therefore, by using (2), the value of the first fault current peak can be calculated by:

(3) $$i_{P}=\frac{V_{m}L\omega }{R^{2}+L^{2}\omega^{2}}e^{-\alpha t_{P}}+\frac{V_{m}}{\sqrt{R^{2}+L^{2}\omega^{2}}}$$

where *i_P_* and *t_P_* are the peak current and time, respectively.

**Figure 3:**
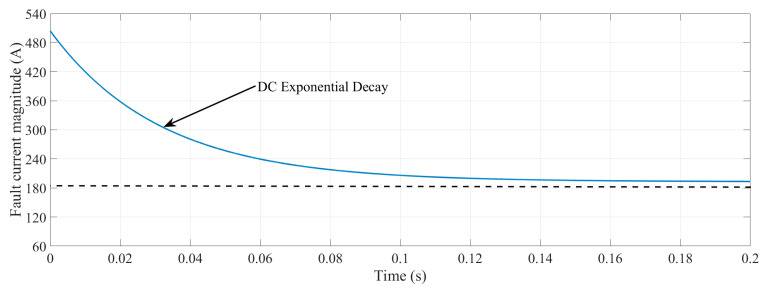
DC decay feature of AC fault currents.

As can be seen in (2), the fault current has a DC decaying transient, which is illustrated in Figure 3. Therefore, it can be observed that the initial moments of fault have the highest magnitude, and it is pivotal to detect and locate faults within the first peaks to avoid any damage to components.

The factor of *L/R* is the time constant of the DC decaying component; in other words, the time constant, *τ*, is the inverse of *α* in (2). By considering the value of *X/R* to be 8 for an MV distribution system, the DC component decays to 45% of its first peak in the second cycle. This characteristic is usable for the rating structure of circuit breakers and shows the importance of locating and detecting faults within the first peaks of the fault current.

## 3. Proposed Fault Location Scheme

**Figure 4:**
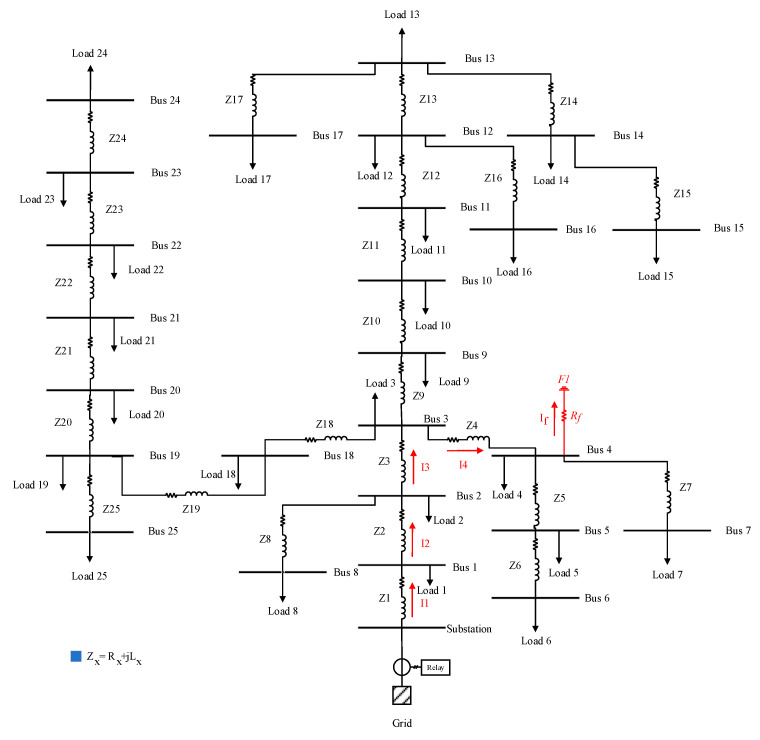
Structure of understudy practical distribution system.

A local transient-based fault location scheme is proposed in this section for a distribution system. The proposed method uses the recorded data of an overcurrent relay at the main feeding point of the distribution system and extracts the fault current features to estimate the fault location. For a practical distribution system, as shown in Figure 4, each line segment has a different line length and type. Therefore, for a fault, *F*1, with the fault resistance of *R_f_*, and fault current of *I_f_*, the voltage–current relationship can be determined by:

(4) $$V_{R}=R_{1}I_{1}+L_{1}\frac{dI_{1}}{dt}+R_{2}I_{2}+L_{2}\frac{dI_{2}}{dt}+R_{3}I_{3}+L_{3}\frac{dI_{3}}{dt}+R_{4}I_{4}+L_{4}\frac{dI_{4}}{dt}+R_{f}I_{f}$$

where *V_R_* is the measured voltage from the relay location, and *I*, *R*, and *L* are the current, resistance, and inductance, respectively, of each line segment, which is specified using subscripts. In distribution systems without any distribution generations, the current value of each line segment can be determined by the difference in current in the upstream line segment and load current of the upstream bus. Therefore, even if it can be assumed that in practical distribution systems the load current consumptions are available and can be collected by smart meters, due to the high sampling time of smart meters being minute, it cannot be directly utilized in transient analysis. Consequently, to propose a local fault location scheme, all current segments are defined based on the main fault current at the relay location and load currents, and so (4) can be rewritten as follows:

(5) $$\begin{array}{l} V_{R}=R_{1}I_{R}+L_{1}\frac{dI_{R}}{dt}+R_{2}(I_{R}-I_{L1})+L_{2}\frac{d(I_{R}-I_{L1})}{dt}+R_{3}(I_{R}-I_{L1}-I_{L2})+\\ +L_{3}\frac{d(I_{R}-I_{L1}-I_{L2})}{dt}+R_{4}(I_{R}-I_{L1}-I_{L2}-I_{L3})+L_{4}\frac{d(I_{R}-I_{L1}-I_{L2}-I_{L3})}{dt}+R_{f}I_{f} \end{array}$$

Consequently, the general equation for the voltage–current relationship for other faulty buses can be defined as:

(6) $$V_{R}=R_{1}I_{R}+L_{1}\frac{dI_{R}}{dt}+R_{f}(I_{R}-\sum_{k=1}^{n-1}I_{Lk})+\sum_{j=2}^{n}R_{j}(I_{R}-\sum_{k=1}^{j-1}I_{Lk})+L_{j}\frac{d(I_{R}-\sum_{k=1}^{j-1}I_{Lk})}{dt}$$

where *n* is the faulty bus number.

Based on (6), the requirement of measuring the fault current of each line is eliminated. Equation (6) can only be used during faults at one bus; however, it can be developed as follows for a fault in a line segment, for example, between buses *n* − 1 and *n*:

(7) $$\begin{array}{l} V_{R}=A_{1}r_{1}I_{R}+A_{1}l_{1}\frac{dI_{R}}{dt}+R_{f}(I_{R}-\sum_{k=1}^{n-1}I_{Lk})+\sum_{j=2}^{n-1}[A_{j}r_{j}(I_{R}-\sum_{k=1}^{j-1}I_{Lk})+A_{j}l_{j}\frac{d(I_{R}-\sum_{k=1}^{j-1}I_{Lk})}{dt}]+\\ +A_{n}r_{n}(I_{R}-\sum_{k=1}^{n-1}I_{Lk})+A_{n}l_{n}\frac{d(I_{R}-\sum_{k=1}^{n-1}I_{Lk})}{dt} \end{array}$$

where *r* and *l* are the resistance and inductance per meter and *A* is the fault distance from bus *n* − 1. On the other hand, the total fault distance from the relay location can be determined by:

(8) $$fault\_location=\sum_{j=1}^{n}A_{j}$$

Therefore, based on the aforementioned concepts, there are two unknown parameters in (7): fault resistance and location. Thus, two equations are necessary to determine these parameters.

During a fault, due to the variation in the resistive characteristic of the distribution system and *X/R*, the difference in the phase angle of current and voltage will also change. Therefore, simply, the new phase angle difference can be calculated by:

(9) $$\arctan(\frac{\omega L_{d}}{R_{d}+R_{f}})=\phi$$

where *L_d_* and *R_d_* are the inductance and resistance between the relay and the faulty point, respectively, and *ϕ* is the phase angle. On the other hand, (3) can be rewritten as:

(10) $$i_{P}=\frac{V_{m}L_{d}\omega }{{\left(R_{f}+R_{d}\right)}^{2}+L_{d}{}^{2}\omega^{2}}e^{-\alpha t_{P}}+\frac{V_{m}}{\sqrt{{\left(R_{f}+R_{d}\right)}^{2}+L_{d}{}^{2}\omega^{2}}}$$

Therefore, by substitution of (9) into (10), the following equation can be determined:

(11) $$i_{P}=(\frac{V_{m}}{L_{d}\omega })\frac{1}{\left(1+\frac{1}{\tan^{2}(\phi )}\right)}e^{-\alpha t_{P}}+\frac{1}{\sqrt{1+\frac{1}{\tan^{2}(\phi )}}}$$

Consequently, by using (11), the inductance from the relay to the faulty point can be calculated, and *L_d_ = A_d_* · *l_d_.* Then, because *l_d_* is known from the type of cable, the fault distance, *A_d_*, can be determined.

To consider, the impact of load current consumption during the fault, it can be assumed that, due to the resistive behavior of loads in distribution systems, the pre-fault and during-fault resistance of these loads will be constant. Therefore, by using the following equation, the pre-fault resistance of the loads can be determined:

(12) $$R_{Loads}=\frac{V_{m}}{\sum I_{Loads}}$$

where *R_Loads_* and *I_Loads_* are the total load resistance and currents in pre-fault conditions. It should be noted that, as smart meters are installed in the load locations of distributed systems and measure the current by minute resolution, the current consumption of loads is available to use in this scheme. During the fault, the new current consumption of loads can be obtained by

(13) $$I_{Loads}^{*}=\frac{V_{m}^{*}}{R_{Loads}}$$

where I*_Loads_ and V*_m_ are the current of loads and voltage during fault. Therefore, (11) can be modified as follows to consider the load impacts:

(14) $$i_{P}^{*}=(\frac{V_{m}^{*}}{L_{d}\omega })\frac{1}{\left(1+\frac{1}{\tan^{2}(\phi )}\right)}e^{-\alpha t_{P}}+\frac{1}{\sqrt{1+\frac{1}{\tan^{2}(\phi )}}}$$

where $i_{P}^{*}$ is the $i_{P}-I_{Load}^{*}$ to use the fault current value for fault location estimation.

## 4. Performance Evaluation

**Figure 5:**
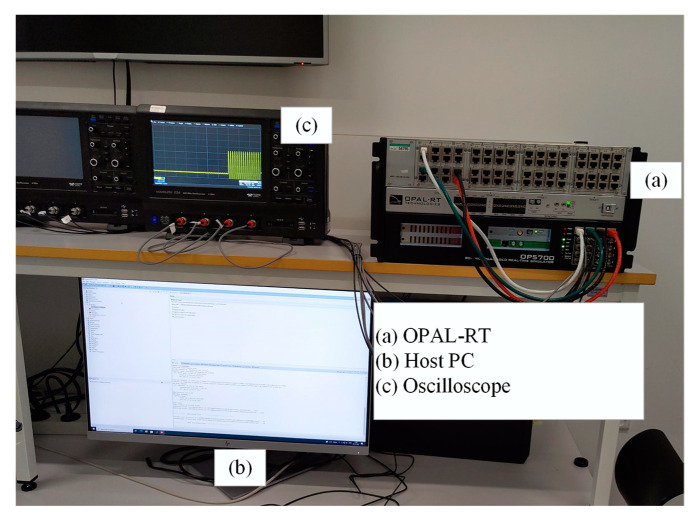
OPAL-RT-based real-time simulation setup.

The test system was constructed in the OPAL-RT real-time simulator OP5700 at Control and Protection in Smart Grids laboratory [21]. The simulator adopted the Red Hat system and its communication through TCP/IP protocol to the monitoring system. In the simulation, the simulation model was first constructed in MATLAB/Simulink, and then the executable model was loaded in the OPAL-RT simulator. The sampling frequency was 10 kHz. The real-time simulation setup is shown in Figure 5.

**Figure 6:**
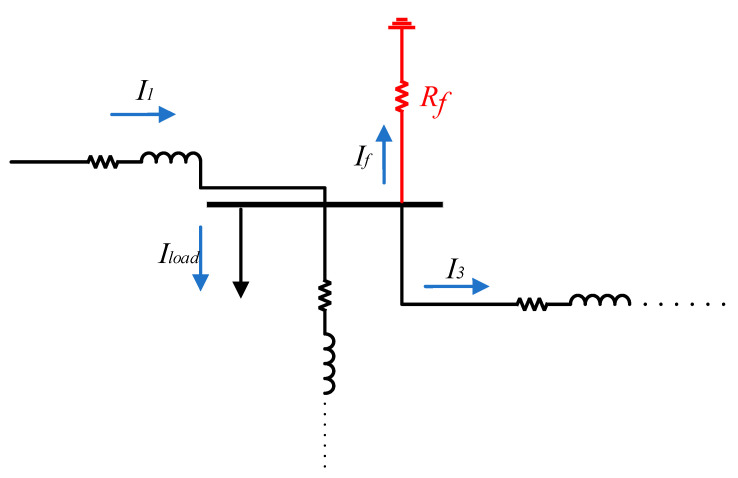
Current distribution during a fault in the distribution system.

The simulated distribution system consists of 25 buses, loads, and lines between them, as shown in Figure 4; hence, the impact of loads cannot be neglected in fault location studies. As shown in Figure 6, loads can consume current during the fault, which should be considered to have an accurate estimation of the fault current at each line branch. In the fault location estimation, the normalized error (*NE*) of calculated fault location is used as follows [22]:

(15) $$NE=\frac{D_{C}-D_{A}}{L_{DS}}\times 100\%$$

where *D_C_* is the calculated fault distance, *D_A_* is the actual fault distance, and *L_DS_* is the line length.

**Figure 7:**
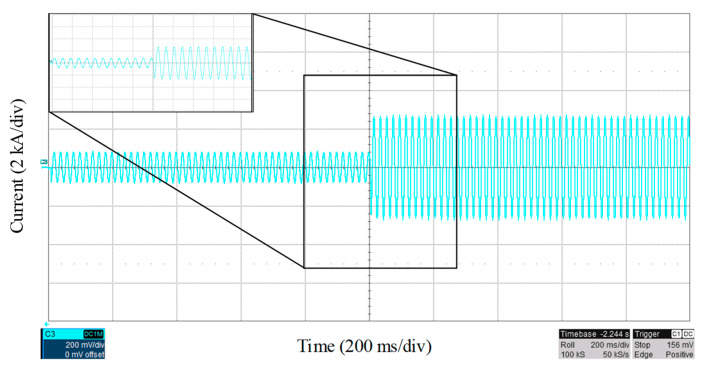
Fault current characteristic with fault at bus 4 with a fault resistance of 1 Ω.

In the fault location techniques, it is vital to locate the fault within a few cycles. Therefore, as the proposed scheme only uses the first peak of the fault current, it can provide a fast fault location scheme. Figure 7 shows the fault current, measured at the mainstream, for a fault at bus 4, as shown in Figure 4, with a fault resistance of 1 Ω. Due to only using the first peak of fault current and the low computational complexity of the proposed method, the fault is located based on the proposed method in Section 3, within 11 ms after the fault event with an accuracy of 94.16%, which shows a very fast operation of the proposed method.

**Figure 8:**
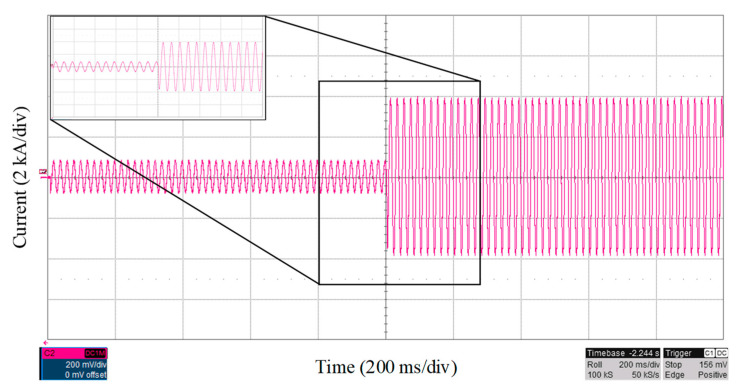
Fault current characteristic with fault at the line between buses 3 and 9 with the fault resistance of 0.2 Ω.

In another scenario, a fault happened at 44% of line 9, between buses 3 and 9, with the fault resistance at 0.2 Ω, and the fault current is shown in Figure 8. The fault is located within 11 ms, with an accuracy of 99.6%.

The estimated fault locations for faults at buses and lines with different fault resistances are shown in Table 1. The value of *NE* will change with different fault locations and resistance scenarios. However, the maximum value of *NE* is limited to 8%, which verifies the accuracy of the proposed method.

It is noteworthy that the proposed method only uses the current and voltage at the grid connection transformer, as shown in Figure 4. Therefore, it is not essential to add any additional equipment or communication links to provide fault location support to the existing distribution system.

**Figure 9:**
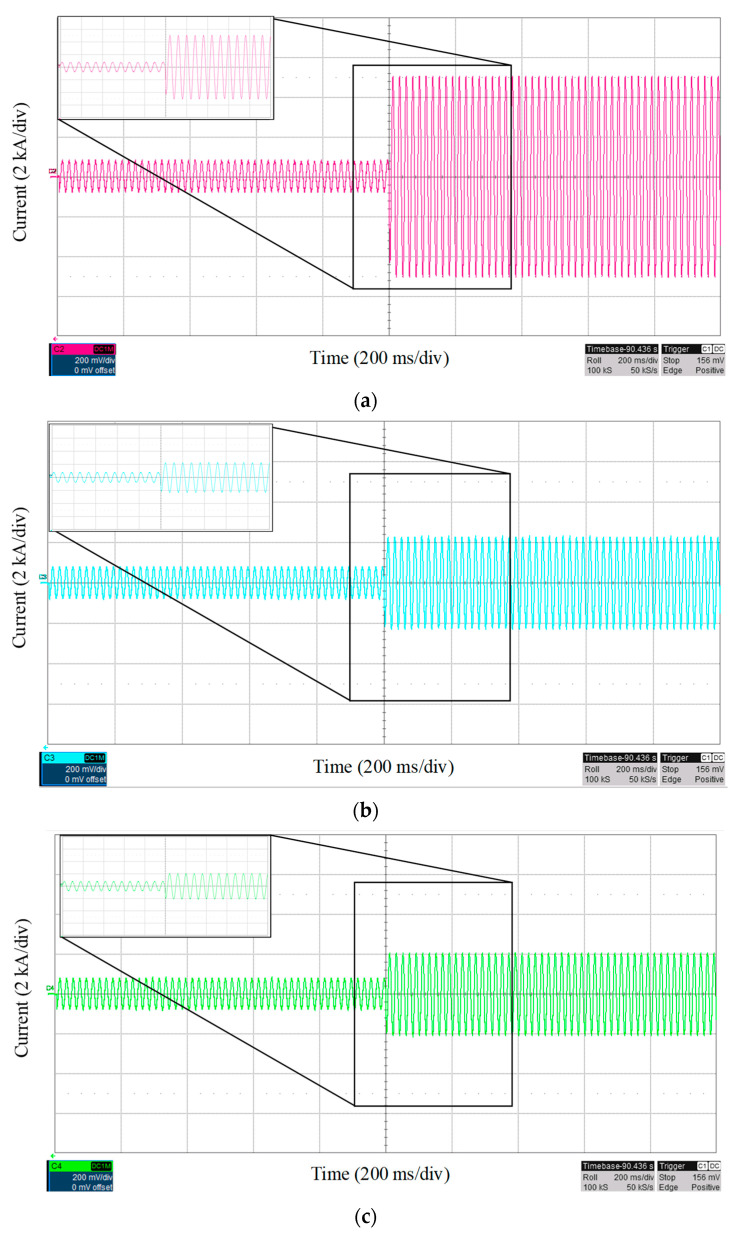
Fault current characteristics with three different fault resistances: (**a**) 0.1 Ω, (**b**) 0.4 Ω, and (**c**) 0.8 Ω.

**Figure 10:**
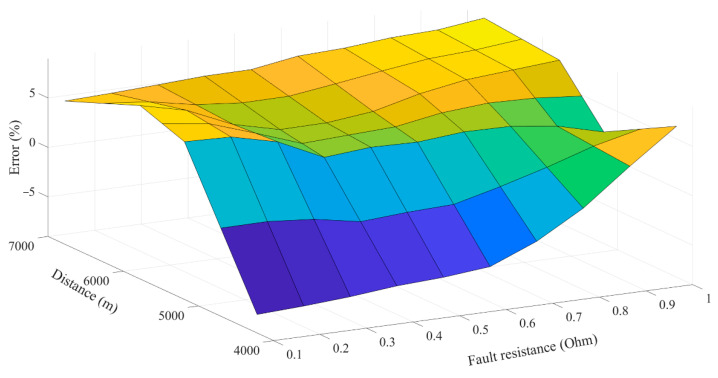
Accuracy evaluation of the proposed scheme.

To evaluate the performance of the proposed fault location scheme in more detail, three different fault resistances are assumed at bus 17, and the fault currents are shown in Figure 9. As expected, by increasing the fault resistance, the value of *NE* will increase. However, as the proposed scheme uses the first peak of fault current, the fault location time will be approximately the same. The evaluation of the proposed method for different fault resistances and locations is presented in Figure 10. The real-time tests are performed for fault resistances up to 1 Ω. As shown in Figure 10, for different fault resistance values, the value of error is limited to a maximum of 8%, which is in an acceptable range.

**Figure 11:**
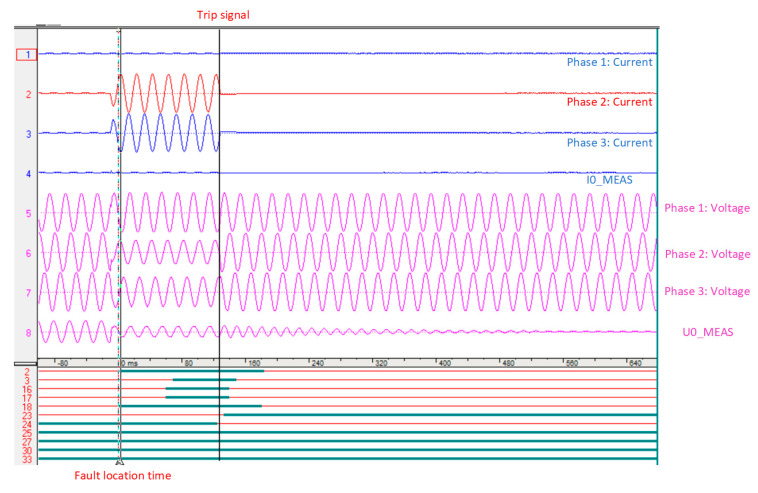
Real recorded fault current and voltage of distribution system.

**Figure 12:**
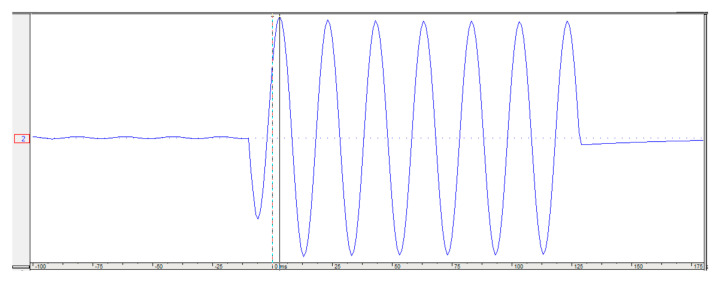
Behavior of a recorded fault current.

To investigate the performance of the proposed method on a real distribution system, the proposed method is applied to real fault current and voltage waveforms recorded by an ABB overcurrent relay, with a sampling rate of 1 kHz, as shown in Figure 11. As can be observed from Figure 11, the usage data are from the time of fault detection to the time of sending the trip signal by overcurrent to the circuit breakers. Therefore, the proposed method is immune against the transients during and after fault isolation by the circuit breaker. The configuration of the understudy distribution system is the same as the structure in Figure 4, and the recorded data are related to a fault event at the line between bus 10 and 11, with a 100 m distance from bus 10, which is a 5286 m distance from the main feeder. As shown in Figure 12 for this real fault, and proved in Section 2, the fault current has the maximum magnitude at the first peak, and due to the DC decaying transients, the next peaks will have lower magnitudes. Before the implementation of the proposed method, the maintenance team used a fault passage indicator located at each of the buses, to aid them in locating the fault. Now, by using the proposed method, the operators will be able to directly check the faulted place instead of visiting every single bus. After using the proposed technique, the fault distance is estimated at a 5065 m distance from the main feeder, which proves 95.84% accuracy of the proposed method on a real fault situation.

As observed from Figure 11 and Figure 12, the proposed method only used the data in the overcurrent relay of the mainstream of a 10 kV system to locate the fault in the distribution system. As shown in Figure 12, the proposed method uses the first peak of the fault current that occurred after 9 ms; therefore, the proposed fault location method can locate the fault quickly. Moreover, due to the low error of the proposed method based on the real data, approximately 4%, the proposed method will indicate the almost exact place of an underground faulty cable.

## 5. Comparison

### 5.1. Quantitative Comparison between the Proposed Method and Distance Relay

The result of the proposed scheme is compared with distance relay, as a well-known fault-locating device in AC power systems [23]. As shown in [24,25,26], in addition to transmission lines, distance relays can be implemented in distribution systems. To accurately compare these two different schemes, a distance relay is implemented in the mainstream of the test case, at the same place as the installed overcurrent relay in Figure 4. Therefore, in the same scenarios as Table 1, the estimated fault distances by using a distance relay are shown in Table 2. As shown in Table 2, by increasing the distance and fault resistance, the accuracy of the traditional distance relays will decrease dramatically, and therefore, these devices are not suitable for fault location applications in distribution systems. Furthermore, because the distance relays use the steady-state fault current, not transient, the operation speed of distance relays is higher than the proposed method. Consequently, the effectivity, accuracy, and operation speed of the proposed scheme are higher than the distance relays.

### 5.2. Qualitative Comparison between the Proposed Method and Existing Fault Location Methods

The results of the proposed method are compared qualitatively with other existing-reported methods in [9], and [27,28,29], as presented in Table 3. In [9], the suggested method requires a fault location device for each line, which makes its implementation too costly and difficult for distribution systems with a high number of branches. Due to using several measurement devices at the distribution system in [27], the cost, noise, and implementation complexity of this method are higher than the proposed local scheme. Moreover, a communication-based method using the Internet of Things is presented in [28] to locate faults in the distribution system. However, this method requires a high number of communication links with high bandwidths, which can be affected by noise and communication delay. The method presented in [29] uses synchronized voltage and current measurements in the distribution system to locate the fault. However, this approach can only locate the faulty component, and therefore, it cannot provide more exact information on the fault location. In contrast, the proposed method is applicable to distribution systems locally, without any communication links and additional units, which reduces the total cost and implementation complexity of the protection system, which might not be acceptable for distribution systems. Moreover, the proposed method can estimate the fault locations very quickly and accurately to ensure fast recovery of the faulty system.

## 6. Conclusions

It has been presented through this research that the local fault location in a distribution system is an unresolved issue, especially in systems with a significant number of branches and different fault resistance values. Implementation of the proposed method, which only requires a current and voltage sensor at the mainstream of the system, will potentially impact the operation, cost, and reliability of distribution systems. A method to locate faults in distribution systems with different fault resistances is proposed in this paper. The method uses the first peak of current and voltage values to locate the fault, and therefore, as an online fault location method, this method provides a fast operation. The proposed method is validated with extensive real-time simulation results on a distribution system. The simulation results indicate that the proposed method gives very satisfactory results even with relatively higher fault resistance values. The fault distance estimation is achieved with an accuracy of 8% in different scenarios. The presented results show the higher accuracy of the proposed scheme. Moreover, in future work, the local fault location of high impedance faults in distribution systems will be investigated.

## Figures and Tables

**Table 1:** Results of estimated fault location for different scenarios.

Faulty Point	Fault Resistance (Ω)	Actual Fault Distance (m)	Estimated Fault Distance (m)	NE (%)
Bus 2	0.1	3421	3472	1.49
Bus 18	0.2	4737	4856	2.51
Bus 19	0.4	5777	6019	4.2
Bus 4	1.0	4278	4528	5.84
Line 18	0.5	4276	4143	−3.1
Line 6	0.6	5399	5816	7.72
Line 7	0.8	4132	4176	1.05

**Table 2:** Results of estimated fault location by distance relay for different scenarios.

Faulty Point	Fault Resistance (Ω)	Actual Fault Distance (m)	Estimated Fault Distance (m)	NE (%)
Bus 2	0.1	3421	3901	14
Bus 18	0.2	4737	5698	20
Bus 19	0.4	5777	7700	33
Bus 4	1.0	4278	9085	112
Line 18	0.5	4276	6679	56
Line 6	0.6	5399	8283	53
Line 7	0.8	4132	7978	93

**Table 3:** Qualitative comparison of the proposed method with other existing methods.

	Method				
Parameter	[9]	[27]	[28]	[29]	Proposed Method
Required external unit	No	Yes	Yes	Yes	No
Communication link	No	Yes	Yes	Yes	No
Error	6%	8%	0.5%	--	8%
Noise and delay	No	Yes	Yes	Yes	No
Cost	Low	High	High	High	Low

## Data Availability

Data sharing not applicable.
